# Thioredoxin-2 Regulates SqrR-Mediated Polysulfide-Responsive Transcription via Reduction of a Polysulfide Link in SqrR

**DOI:** 10.3390/antiox12030699

**Published:** 2023-03-11

**Authors:** Takayuki Shimizu, Masaru Hashimoto, Tatsuru Masuda

**Affiliations:** Graduate School of Arts and Sciences, The University of Tokyo, Tokyo 153-8902, Japan

**Keywords:** polysulfide, transsulfuration, redox signaling

## Abstract

Polysulfide plays an essential role in controlling various physiological activities in almost all organisms. We recently investigated the impact of polysulfide metabolic enzymes on the temporal dynamics of cellular polysulfide speciation and transcriptional regulation by the polysulfide-responsive transcription factor SqrR in *Rhodobacter capsulatus*. However, how the polysulfidation of thiol groups in SqrR is reduced remains unclear. In the present study, we examined the reduction of polysulfidated thiol residues by the thioredoxin system. TrxC interacted with SqrR in vitro and reduced the polysulfide crosslink between two cysteine residues in SqrR. Furthermore, we found that exogenous sulfide-induced SqrR de-repression during longer culture times is maintained upon disruption of the *trxC* gene. These results establish a novel signaling pathway in SqrR-mediated polysulfide-induced transcription, by which thioredoxin-2 restores SqrR to a transcriptionally repressed state via the reduction of polysulfidated thiol residues.

## 1. Introduction

Polysulfide modulates a variety of physiological functions, potentially by acting as a signaling molecule. Polysulfidation of electrophilic species and thiol residues in a protein is reportedly critical for polysulfide-mediated signal transduction [1,2,3,4]. In mammals, electrophilic thiolation of 8-nitroguanosine 3′,5′-cyclic GMP (which accumulates in cells under nitrosative stress) via attack by a hydropersulfide blocks protein *S*-guanylation, thus modulating redox signaling [1,2]. Polysulfidated proteins have been comprehensively analyzed in both mammals and plants, in which a small but significant fraction of the proteome is polysulfidated [5,6,7,8]. Diverse bacteria may also provide bioavailable mobile sulfur to the organism [9].

We recently characterized the dynamics of polysulfide metabolism with regard to bacterial polysulfide-responsive transcription in *Rhodobacter capsulatus* [10]. SqrR (rcc01453), a bacterial polysulfide sensor isolated from *R. capsulatus*, exerts extensive control over sulfide-responsive genes that encode polysulfide metabolism-related proteins in *R. capsulatus* [11]. SqrR forms an intramolecular polysulfide crosslink via two conserved Cys residues when exposed to polysulfide, resulting in a decline in repressor activity [10,11]. A mass spectrometry-based kinetic profiling study further defined this polysulfidation process and the chemical specificity of SqrR [12]. These data indicate that SqrR-related polysulfide signal transduction is a suitable model system for investigations of sulfide/polysulfide signaling. Our current study revealed that two SqrR-regulated polysulfide-metabolizing enzymes, sulfide:quinone reductase (SQR) (rcc00785) and rhodanese (rcc01557), affect SqrR-mediated polysulfide-induced transcription and speciation of intracellular polysulfide, which in turn modulates the polysulfide response in *R. capsulatus* [10]. SQR provides sustained levels of polysulfide to suppress the transcriptional repression caused by the reduction of SqrR. Moreover, rhodanese appears to decrease the polysulfidated state of SqrR via polysulfide reduction by intermolecular transsulfuration. However, how the polysulfidation of SqrR is directly abolished remains unclear.

A number of studies have described the contribution of thioredoxin to the reduction of inorganic polysulfide and protein persulfide in mammals and bacteria [6,13,14,15,16,17]. Mammalian thioredoxins exhibit *S*-desulfhydrase activity, which catalyzes the *S*-desulfhydration of the active site persulfide-formed cysteine(s) of 3-phosphate dehydrogenase and pyruvate carboxylase [14]. Moreover, thioredoxin/thioredoxin reductase-mediated *S*-desulfhydration reduces polysulfidated caspase in the inactivated state, thereby suppressing apoptosis [13]. Bacterial thioredoxins also reduce protein persulfides, which control critical metabolic and regulatory mechanisms under conditions of sulfide/polysulfide stress [15,18]. In addition, thioredoxin mediates the transsulfuration reaction between protein-bound persulfide intermediates during Fe-S cofactor biogenesis [16].

Interestingly, RNA-seq data from our previous study indicated that the transcription of thioredoxin-2 (TrxC) is regulated by SqrR in response to sulfide [11]. Here, we provide evidence that TrxC regulates SqrR-mediated polysulfide-induced transcription via depolysulfidation of thiol residues in SqrR.

## 2. Materials and Methods

### 2.1. Bacterial Strains, Media, and Growth Conditions

*Rhodobacter capsulatus* strain SB1003 and mutant strains were grown aerobically at 30 °C in a PYS medium [19]. The medium was supplemented with gentamycin and rifampicin at concentrations of 1.5 µg/mL and 75 µg/mL, respectively.

*Escherichia coli* strains were cultured aerobically in Luria Bertani (LB) medium at 37 °C. The medium was supplemented with ampicillin and gentamycin concentrations of 100 µg/mL and 10 µg/mL, respectively.

### 2.2. Overexpression and Purification of SqrR and TrxC

Recombinant SqrR-FLAG and His-tagged TrxC were overexpressed in *E. coli* strain BL21 (DE3) utilizing a previously described [11] pSUMO::SqrR-FLAG plasmid and pColdI::TrxC plasmid, respectively. To construct pColdI::TrxC, a DNA fragment encoding full-length *trxC* was amplified by polymerase chain reaction (PCR) using KOD One polymerase (TOYOBO) and the TrxC-F and TrxC-R primers (Table 1). The resulting amplified DNA was cloned into the *Nde*I-cut pColdI vector using an In-Fusion HD Cloning kit (Clontech). Overexpression of the recombinant proteins was induced by the addition of 0.2 mM isopropyl-β-D-thiogalactopyranoside and incubation at 16 °C overnight (16–18 h). Cells in a 500-mL culture were harvested and stored at −80 °C until further use. SrqR-FLAG was purified as previously described [11]. TrxC was purified using a 1-mL HisTrap column and ÄKTA Start system (Cytiva). Bacteria were resuspended in 20 mL of cell buffer composed of 20 mM Tris-HCl (pH 8.0), 500 mM NaCl, 5 mM imidazole, and 10% glycerol and then lysed by sonication. The lysate was clarified by centrifugation at 30,000× *g* for 30 min at 4 °C, and the supernatant was filtered using a 45-µm membrane filter (Millipore). The resulting lysate was loaded onto a HisTrap column and washed with 20 column volumes of wash buffer consisting of 20 mM Tris-HCl (pH 8.0), 500 mM NaCl, 20 mM imidazole, and 10% glycerol. TrxC was eluted with a gradient of 20 mM to 500 mM imidazole in the loading buffer over a total of 10 column volumes. Protein concentration was determined using the Bradford assay.

### 2.3. Pull-Down Assay

Recombinant SqrR-FLAG and TrxC were dialyzed against a wash buffer consisting of 20 mM Tris-HCl (pH 8.0), 500 mM NaCl, 20 mM imidazole, and 10% glycerol. Ni-resin and protein (5 μM) were mixed and incubated for 3 h at 4 °C. After incubation, Ni-NTA agarose (QIAGEN) and the protein mixture were transferred to a poly-prep chromatography column (Bio-Rad) and washed with 20 column volumes of wash buffer. Proteins were eluted with 1 mL of 500 mM imidazole-containing elution buffer. The eluates were analyzed by Western blotting using an anti-FLAG antibody, as described previously [11].

### 2.4. Analysis of the Redox State of Cysteine Thiols

Recombinant SqrR-FLAG and TrxC were reduced by incubation with 0.5 mM dithiothreitol (DTT) for 60 min at room temperature. After reduction, DTT was removed by ultrafiltration in an anaerobic glove box using a degassed buffer consisting of 25 mM Tris-HCl (pH 8.0) and 200 mM NaCl. Reduced SqrR-FLAG was anaerobically incubated with a 50-fold molar excess of glutathione persulfide (GSSH) for 30 min at room temperature, and unreacted GSSH was removed using the same method used for DTT removal. GSSH-treated SqrR-FLAG was mixed anaerobically with the same molar excess of TrxC and incubated for 30 min at room temperature. A 100-μL volume of each SqrR sample was adjusted to 10 μM, mixed with 10 μL of 100% trichloroacetic acid (TCA), and incubated on ice for 20 min. Proteins were precipitated by centrifugation at 20,000× *g* and then washed with cold acetone to remove the TCA. The precipitates were resuspended in 50 µL of a buffer consisting of 1% SDS, 50 mM Tris-HCl (pH 7.5), and 0.1 mM polyethylene glycol (PEG)-maleimide. A PEG-maleimide modification was performed at 37 °C for 30 min. The resulting proteins were separated on 10% SDS-PAGE gels, and SqrR-FLAG was specifically detected by Western blotting using an anti-FLAG antibody.

### 2.5. Cloning and Mutagenesis

The plasmid pZJD29a::Δ*trxC* was used to disrupt *trxC* in *R. capsulatus*, as previously described [11]. Two ~500-bp DNA fragments encoding the N- and C-terminal regions of *trxC* were amplified by PCR using KOD One polymerase (TOYOBO). Two sets of primer pairs were used for the amplification: one pair consisting of the forward primer trxC F1 and reverse primer trxC R1, and the other pair consisting of the forward primer trxC F2 and reverse primer trxC R2 (Table 1). The two fragments were cloned into the *Bam*HI-site in pZJD29a [20] using an In-Fusion HD Cloning kit (Clontech). The resulting plasmids were introduced into *R. capsulatus* by conjugation with *E. coli* strain S17-1/*λpir*, and a subsequent homologous recombination event was induced as described in a previous report [20]. The deletion was confirmed in the isolated mutants by sequencing analysis. For the construction of the complementing strain of *trxC* mutant, full-length trxC containing the 500-bp upstream and downstream regions of *trxC* fused FLAG sequence at the 3′-end of *trxC* was amplified by PCR and cloned into the *Bam*HI-site in pZJD3 [21]. The resulting plasmid was introduced into *R. capsulatus* Δ*trxC* mutant cells as described above. Subsequent single–cross-over recombinants were isolated as *trxC* complementing strain.

### 2.6. RNA Isolation and Quantitative Real-Time PCR (qRT-PCR)

*Rhodobacter capsulatus* was cultured aerobically to the log phase or stationary phase. For sulfide treatment, Na_2_S at a final concentration of 0.2 mM was added when the cells reached the mid-log phase (OD_660_ = 0.7), and the cells were then cultured further. Aliquots of 0.5 mL of cells were harvested at each time point (0, 2, 30, 60, 120 min), and total RNA was extracted from each sample using NucleoSpin RNA Plus (TaKaRa). The quality of purified RNA was assessed based on a typical OD_260_ to OD_280_ ratio of approximately 2.0. The RNA was reverse transcribed using a PrimeScript RT Reagent kit (TaKaRa), and qRT-PCR assays were performed using THUNDERBIRD Next SYBR qPCR mix (TOYOBO) and a CFX Connect Real-Time system (Bio-Rad). The housekeeping gene *rpoZ*, which encodes RNA polymerase, was analyzed as an internal control using gene-specific primers (Table 1).

## 3. Results

### 3.1. Identification of TrxC

To verify whether thioredoxin is involved in transcriptional regulatory signaling by SqrR, we utilized the previous RNA-seq transcriptomic data of *R. capsulatus* WT and Δ*sqrR* in the absence and presence of exogenous sulfide [11]. Transcription of *trxC* gene encoding thioredoxin-2 (rcc02517) was up-regulated more than 20-fold by both treatments with exogenous sulfide and by disruption of *sqrR* (Table 2). This gene is located in a different position on the chromosome from *sqr*. Based on this observation and in consideration of the molecular functions of thioredoxin, it appears that TrxC plays a role in reducing the polysulfide crosslink in SqrR.

### 3.2. Interaction between SqrR and TrxC

**Figure 1:**
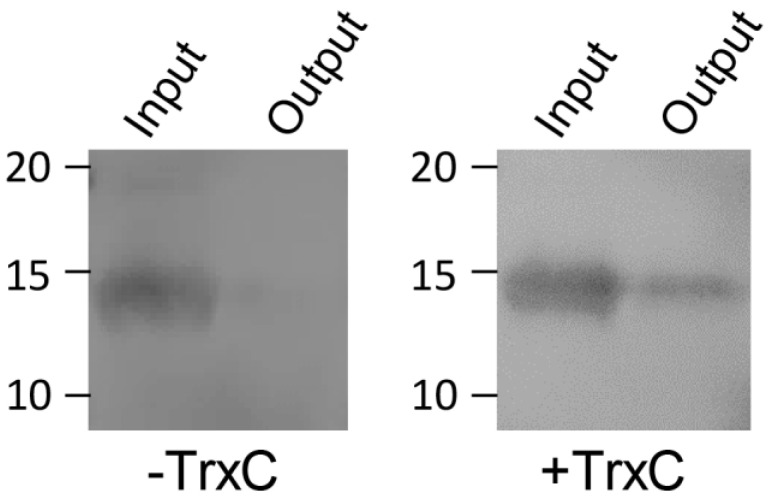
His-tag–based pull-down assay of the SqrR–TrxC interaction. SqrR-FLAG was mixed with Ni-resin in the absence (−TrxC) and presence (+TrxC) of His-tagged TrxC. Input and output were analyzed by Western blotting using an anti-FLAG antibody to detect SqrR-FLAG. The numbers to the left of the images indicate molecular weight based on size markers (kDa).

A pull-down assay using recombinant FLAG-tagged SqrR and His-tagged TrxC was performed to determine whether TrxC interacts with SqrR. Briefly, FLAG-tagged SqrR was mixed with His-tagged TrxC–bound Ni-resin and co-eluted after extensive washing of the resin. FLAG-tagged SqrR was specifically detected by Western blotting using an anti-FLAG antibody (Figure 1), as the molecular weights of SqrR-FLAG and TrxC are similar. FLAG-tagged SqrR did not bind to the Ni-resin in the absence of TrxC but did co-elute with TrxC. This result indicated that a positive interaction occurs between TrxC and SqrR in vitro.

**Figure 2:**
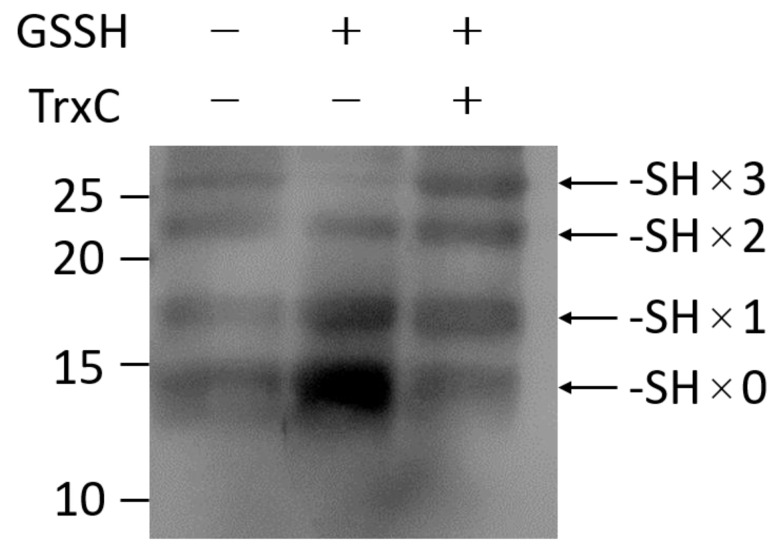
Shift in SqrR SDS-PAGE mobility caused by thiol modification. Reduced, GSSH-treated, and GSSH- and TrxC-treated SqrR-FLAG samples were labeled with PEG-maleimide. Each PEG-maleimide-modified SqrR was detected by Western blotting using an anti-FLAG antibody. Labels to the right of the image indicate the number of modified cysteine thiols. The numbers to the left of the image indicate molecular weight based on size markers (kDa).

SqrR forms an intramolecular polysulfide crosslink between two cysteine residues following polysulfide exposure [10,11]. We, therefore, analyzed the role of TrxC in the reduction of the polysulfide crosslink in SqrR. Reduced SqrR was treated with a 50-fold molar excess of GSSH relative to the concentration of free protein subunit, and any remaining free thiol residues were modified by treatment with PEG-maleimide under anaerobic conditions. PEG-maleimide–modified SqrR species were separated by SDS-PAGE to identify completely reduced SqrR and crosslinked SqrR. As SqrR has three Cys residues (C9, C41, C107), four different bands were detected (Figure 2). In the case of reduced SqrR, a band derived from SqrR was detected in which the thiol group was completely reduced (top band). This top band disappeared after reduced SqrR was treated with GSSH, and the intensity of a band with two thiol groups protected from modification by PEG-maleimide (third band from the top) was increased instead, indicating the presence of an intracellular polysulfide crosslink between two thiols. In contrast, when GSSH-treated SqrR was incubated with reduced TrxC under anaerobic conditions, the intensity of the band derived from crosslinked SqrR decreased, and the band pattern was similar to that of reduced SqrR. These data suggest that TrxC reduces the polysulfide crosslink to thiol groups in SqrR.

### 3.3. Effect of TrxC on SqrR-Mediated Transcription

**Figure 3:**
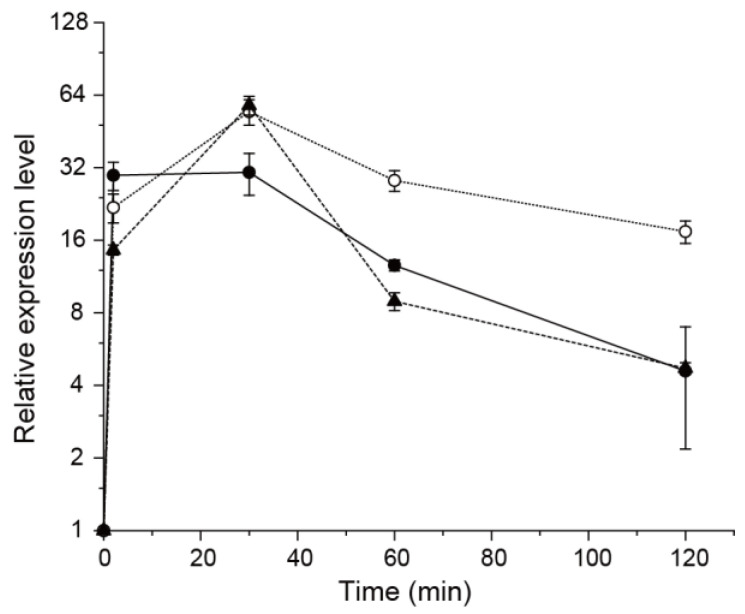
Responsiveness of SqrR regulated gene to sulfide. Temporal changes in the relative level of *sqr* gene transcripts after treatment with sulfide compared with 0 min in WT (filled circles), Δ*trxC* (open circles), and *trxC* complementing strain (filled triangles) cells. Cells were cultured to the mid-log phase under aerobic conditions, and 0.2 mM sodium sulfide was added at t = 0. Cells were harvested at each time point and assayed by qRT-PCR. Data shown are mean ± S.E. from three biological replicates (error bars).

To examine the effect of TrxC on SqrR-mediated polysulfide-induced transcription, we generated deletion mutants and monitored expression levels of the SqrR-regulated gene *sqr*. After treatment with sulfide, the WT strain showed a rapid increase in *sqr* transcript levels, followed by a gradual decrease and, at later time points, a sustained high level of expression relative to that before treatment (Figure 3). In contrast, compared with the WT, the *trxC*-deletion mutant (Δ*trxC*) increased the duration of *sqr* expression after sulfide induction (Figure 3). Furthermore, the *trxC* complementing strain showed similar transcriptional changes as WT. In our previous study, rhodanese (rcc01557)-deletion mutant maintained high expression levels of *sqr* at longer time points as well [10]. These observations are thought to be due to abnormal degradation of polysulfidation in SqrR and intracellular polysulfide. These data suggest that TrxC contributes to the abolition of SqrR-mediated polysulfide-induced transcription.

## 4. Discussion

We studied the contribution of TrxC to the polysulfidation of SqrR and SqrR-mediated polysulfide-induced transcription to explore the possibility of a novel regulatory process in polysulfide signal transduction. We demonstrate that TrxC reduces the intramolecular polysulfide crosslink between two cysteine residues in SqrR and restores SqrR to a reduced transcriptional repression mode. This conclusion is based on the effect of recombinant TrxC on the redox state of thiol residues in SqrR (Figure 2) and the effect of *trxC* deletion on transcriptional changes in SqrR-regulated genes (Figure 3). The in vitro reaction of polysulfidated SqrR with reduced TrxC clearly inhibited the crosslinking between thiol residues in SqrR (Figure 2). Consistent with this biochemical response, Δ*trxC* did not restore SqrR-mediated repression compared with the WT (Figure 3).

Polysulfide was recently identified as an important factor in controlling intracellular redox homeostasis and metabolic regulation [22,23,24], but high concentrations of polysulfide are toxic to cells [25,26]. Thus, as polysulfide exhibits both harmful and beneficial effects, organisms must strictly control intracellular polysulfide levels to leverage the beneficial effects while avoiding cytotoxicity. Thioredoxin-based polysulfide homeostasis may be one of the key regulatory mechanisms in polysulfide signaling. Indeed, the mammalian thioredoxin system enhances survival in the presence of toxic amounts of inorganic polysulfide [6]. Similarly, transcriptional regulation mediated by TrxC plays a role in maintaining polysulfide homeostasis. Our previous study revealed that SQR is de-repressed in the presence of sulfide and generates polysulfide, thereby maintaining the polysulfide modification of SqrR to keep it in a de-repressed state [10]. However, this transient sulfide-stimulated enhanced transcription returns to a transcriptionally repressed state within a few hours. Although not evaluated in detail in the present study, one possible explanation is that polysulfide metabolism mediated by rhodanese plays a role in preventing a continued rise in SQR-derived polysulfide levels. Thus, the TrxC system might contribute to the sulfide-induced maintenance of polysulfide homeostasis via direct reduction of SqrR. Indeed, in *E. coli*, TrxC, which is regulated in response to sulfide by OxyR, has sulfide-induced reducing activity toward intracellular polysulfides [27].

**Figure 4:**
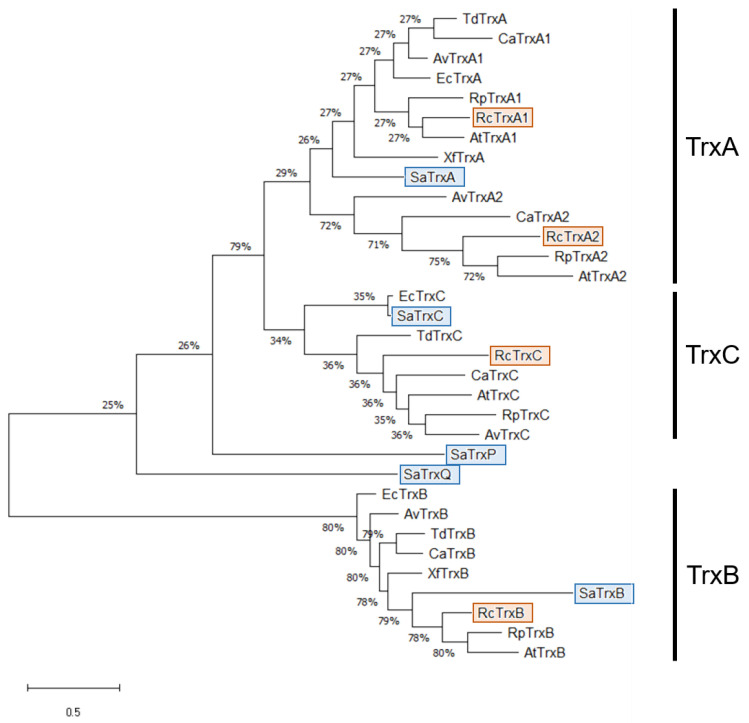
Phylogenic tree based on amino acid sequences of TrxA, TrxC, TrxP, TrxQ, and TrxB homologs. Red and blue boxes indicate the genes of *R. capsulatus* and *S. aureus*, respectively. Phylogenetic analysis was performed using the ClustalX [34] and MEGA [35] programs. The tree was generated using the maximum parsimony method. The first two letters of the protein name indicate the bacterium: Rc, *Rhodobacter capsulatus*; Sa, *Staphylococcus aureus*; Td, *Thiobacillus denitrificans*; Ca, *Comamonas aquatica*; Av, *Allochromatium vinosum*; Ec, *Escherichia coli*; Rp, *Rhodopseudomonas palustris*; At, *Agrobacterium tumefaciens*; Xf, *Xylella fastidiosa*. Sequences of TrxB proteins were used as the outgroup. Accession numbers of each gene are as follows; EcTrxA (WP_097403417), EcTrxB (WP_097680097), EcTrxC(WP_096099216), RcTrxA1(WP_013065783), RcTrxA2(WP_013069030), RctrxB(WP_013068521), RcTrxC(WP_136904981), SaTrxA(WP_001018930), Sa-TrxB(WP_000134958), SaTrxC(NGC70079), SaTrxP(WP_162635110), SaTrxQ(WP_117231667), RpTrxA1(WP_011439531), RpTrxA2(WP_011500882), RpTrxB(WP_044414730), RpTrxC(WP_107357355), AvTrxA1(WP_012969831), AvTrxA2(WP_200157501), AvTrxB(WP_012971465), AvTrxC(WP_200157500), TdTrxA(WP_011310549), TdTrxB(WP_059756818), TdTrxC(WP_018078157), CaTrxA1(WP_042416164), CaTrxA2(WP_043378462), CaTrxB(WP_219163860), CaTrxC(WP_042417992), AtTrxA1(WP_042615683), AtTrxA2(WP_112358989), AtTrxB(WP_112360347), AtTrxC(QCM14208), XfTrxA(WP_004084795), XfTrxB(WP_004089132).

In *Staphylococcus aureus*, two novel thioredoxin-like proteins, TrxP and TrxQ, and the canonical thioredoxin, TrxA, play roles in maintaining polysulfide homeostasis [15,18]. Although these three thioredoxins are bona fide sulfurtransferases, they do not share the most common candidate substrates [15]. In particular, TrxP exhibits greater catalytic efficiency and recognizes more candidate substrates than the other thioredoxins, indicating that TrxP is the primary regulator of polysulfide shuttling in this bacterium. *Rhodobacter capsulatus* expresses three thioredoxin proteins, TrxA1, TrxA2, and TrxC, two of which, TrxA1 and TrxA2, are not regulated by SqrR [11]. These three thioredoxins harbor the canonical WCGPC active site [28], whereas SaTrxP harbors a WCPDC active site [15]. Moreover, RcTrxA1 and A2, RcTrxC, and SaTrxP form phylogenetically different clades (Figure 4). Given that *R. capsulatus* does not harbor a SaTrxP homolog, TrxC is probably the primary regulator maintaining polysulfide homeostasis in the SqrR-mediated polysulfide response in this bacterium.

Studies of the functions of TrxA and TrxC in the oxidative stress response in *E. coli* revealed that these two thioredoxins exhibit equivalent functions in most oxidative stress responses, although their mechanisms of transcriptional regulation differ [29,30]. Thioredoxins play important roles in not only the oxidative stress response [31] but also the oxygen-dependent regulation of photosynthesis genes in *R. capsulatus* and the phylogenetically closely related bacterium *R. sphaeroides* [32,33]. In contrast, TrxA and TrxC exert opposite effects in the regulation of photosynthetic gene expression, because reduced TrxA and oxidized TrxC exert positive and negative effects, respectively, on the DNA supercoiling activity of DNA gyrase. Although TrxC is a thioredoxin secondary to TrxA in bacteria, deletion of *trxC* clearly suppressed the reduction in the transcript at a longer time point (Figure 3), implying that TrxC functions at least as an SqrR-induced polysulfide homeostasis system. Thus, although TrxA and TrxC play essentially redundant physiological roles, each also exerts specific functions. Despite the functional differences between TrxC and TrxA and the metabolic influence and degree to which TrxC is part of a regulatory cascade in SqrR-mediated polysulfide-induced transcription (which are not yet fully understood), the results of the present study expand understanding of the biological significance of the bacterial thioredoxin system in polysulfide signaling.

## 5. Conclusions

Our data suggest that TrxC functions as an “off-switch” to restore SqrR-mediated transcriptional repression. Although TrxC appears to reduce the polysulfidation of thiol residues in SqrR, details regarding the molecular kinetics of this depersulfidation process remain unclear. However, our discovery of TrxC as a novel mediator of polysulfide signaling should facilitate further elucidation of the entire regulatory network in this model (poly)sulfide-responsive bacterium.

## Figures and Tables

**Table 1:** List of primers used in this study.

Name (Accession Number)	Sequence 5′–3′	Purpose
TrxC-F (ADE86247)	TCGAAGGTAGGCATATGATGGGGGCCAAGATGGCG	Overexpression of recombinant protein
TrxC-R	GTACCGAGCTCCATATCAGGCGCGGGCGCCCAGCTTGCCG	
trxC F1	CGACTCTAGAGGATCAAAGATCGGCAGCCGCATCGGCATCTC	Gene disruption
trxC R1	CTTGGCCCCCATCATATTCGCGTTGCGGAATATAT	
trxC F2	ATGATGGGGGCCAAGGGCGCCCGCGCCTGAGAACCCGCGC	
trxC R2	CGGTACCCGGGGATCCCGGCAGGCGTCGCCGACGAAATCGACCGC	
*rpoZ* qF (ADE87042)	GAGATCGCCGATGAAACC	qRT-PCR
*rpoZ* qR	TCGTCGACCTCGATCTGG	
*sqr* qF (ADE84550)	CGCAAGGAAGACAAGGTCAC	
*sqr* qR	CGAGGGCACGAAATGATAC	

**Table 2:** Effects of sulfide and loss of SqrR on levels of *trxC* gene transcription in WT bacteria. Data are cited from [11].

Accession Number	Fold-Change ± lfcSE (with/without Sulfide)	Fold-Change ± lfcSE (Δ*sqrR*/WT)
rcc02517	22.4 ± 1.2	21.6 ± 1.3

## Data Availability

Not applicable.
